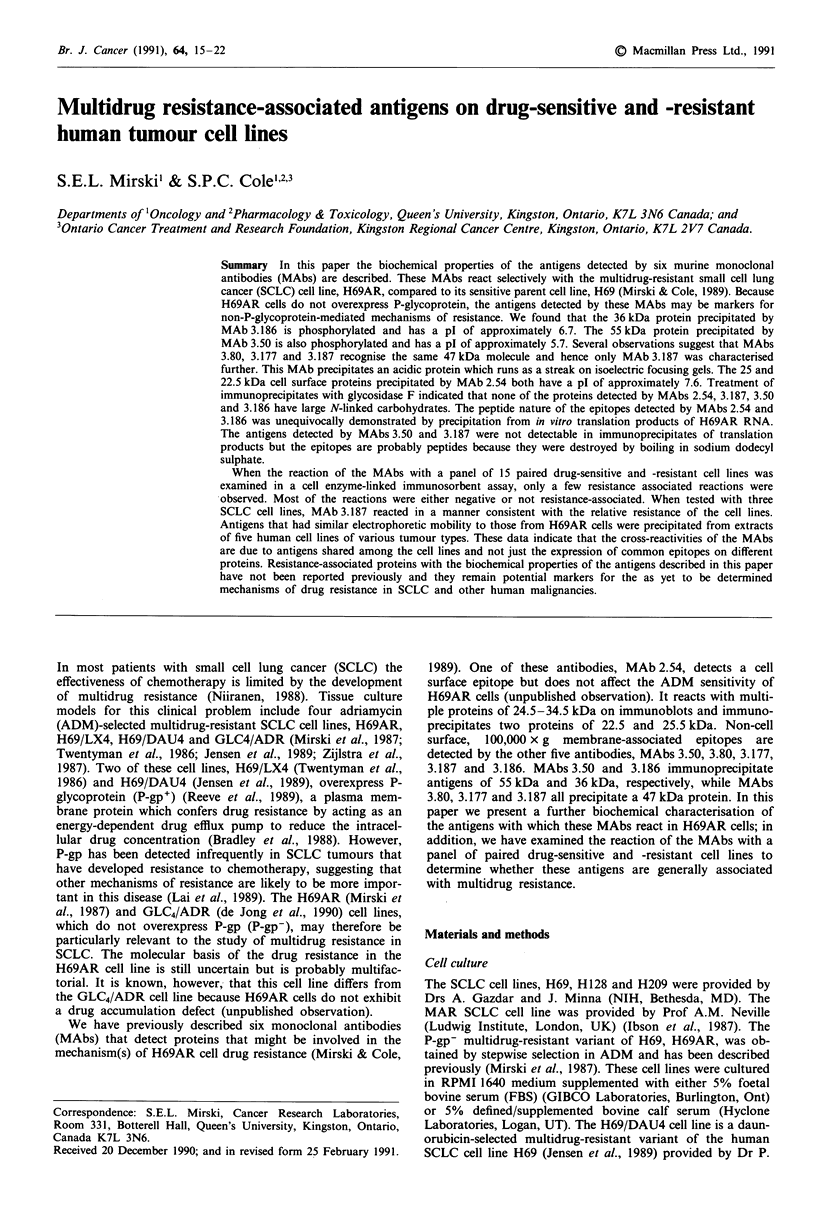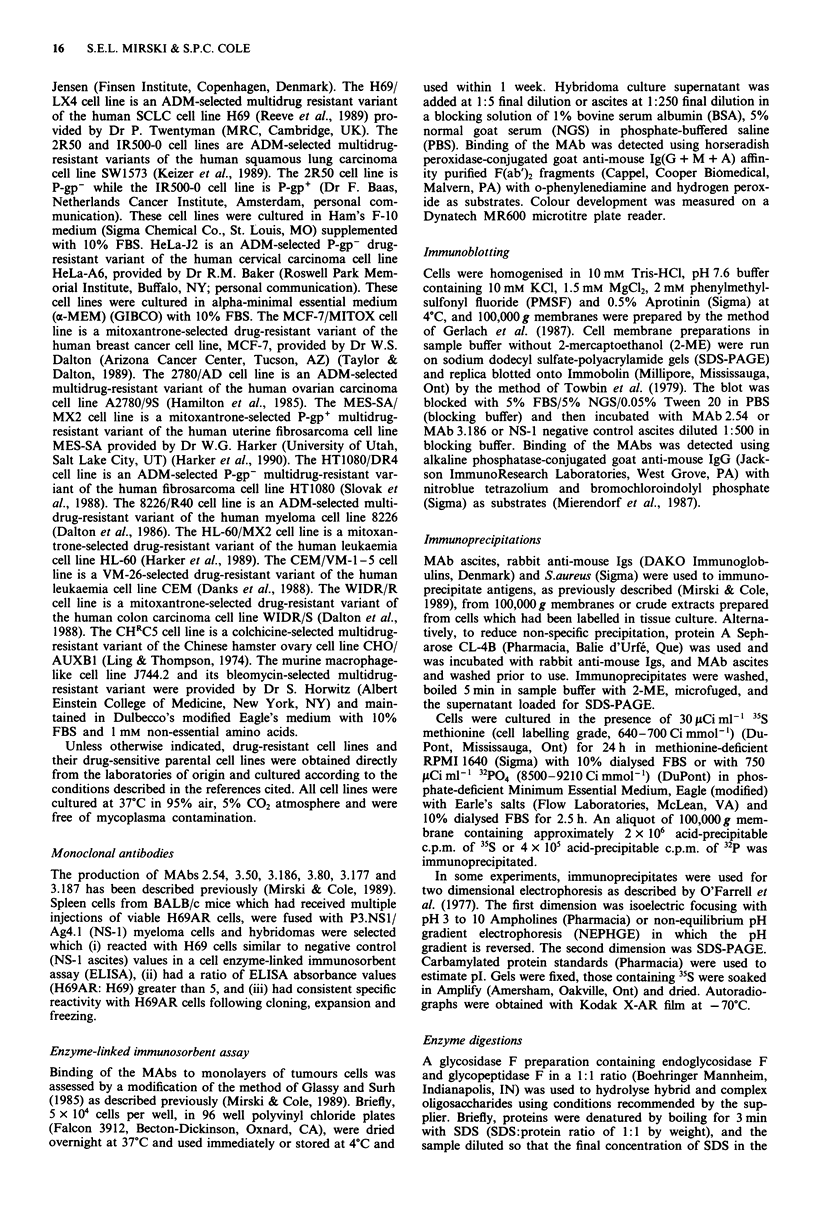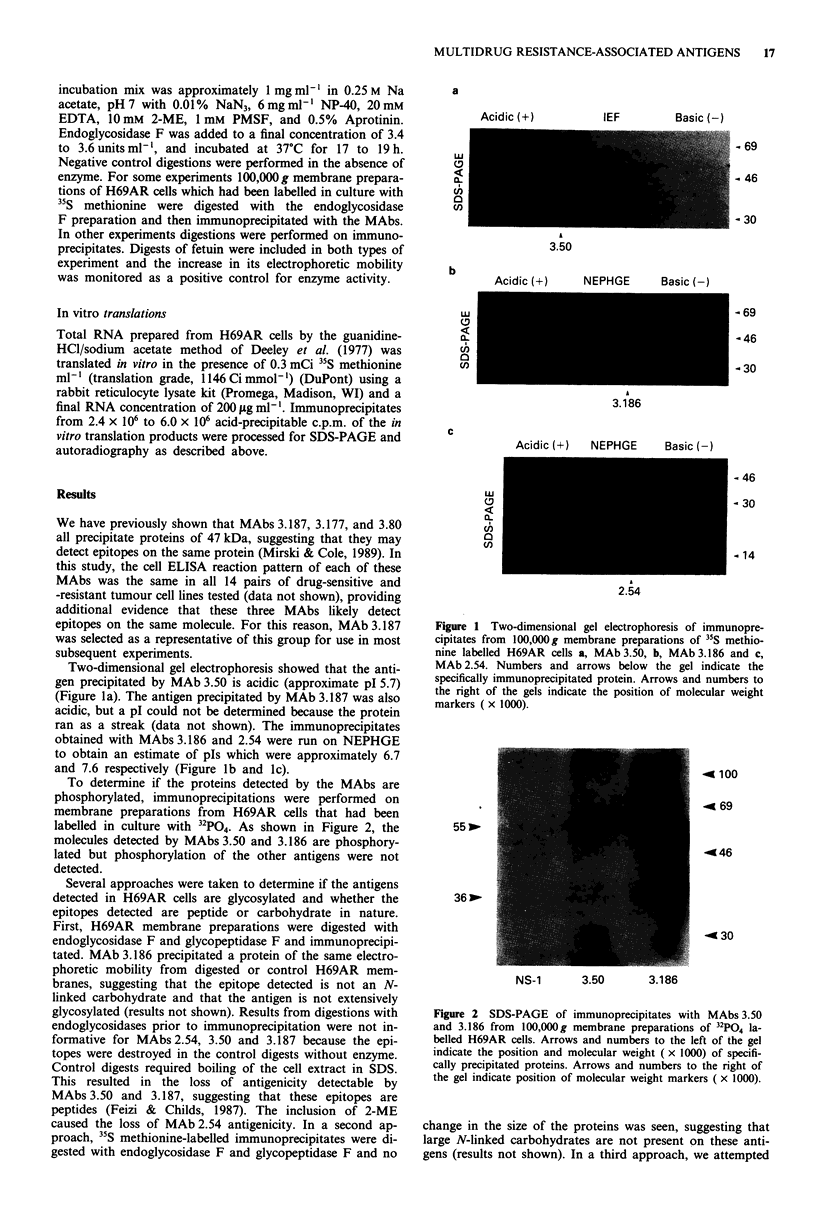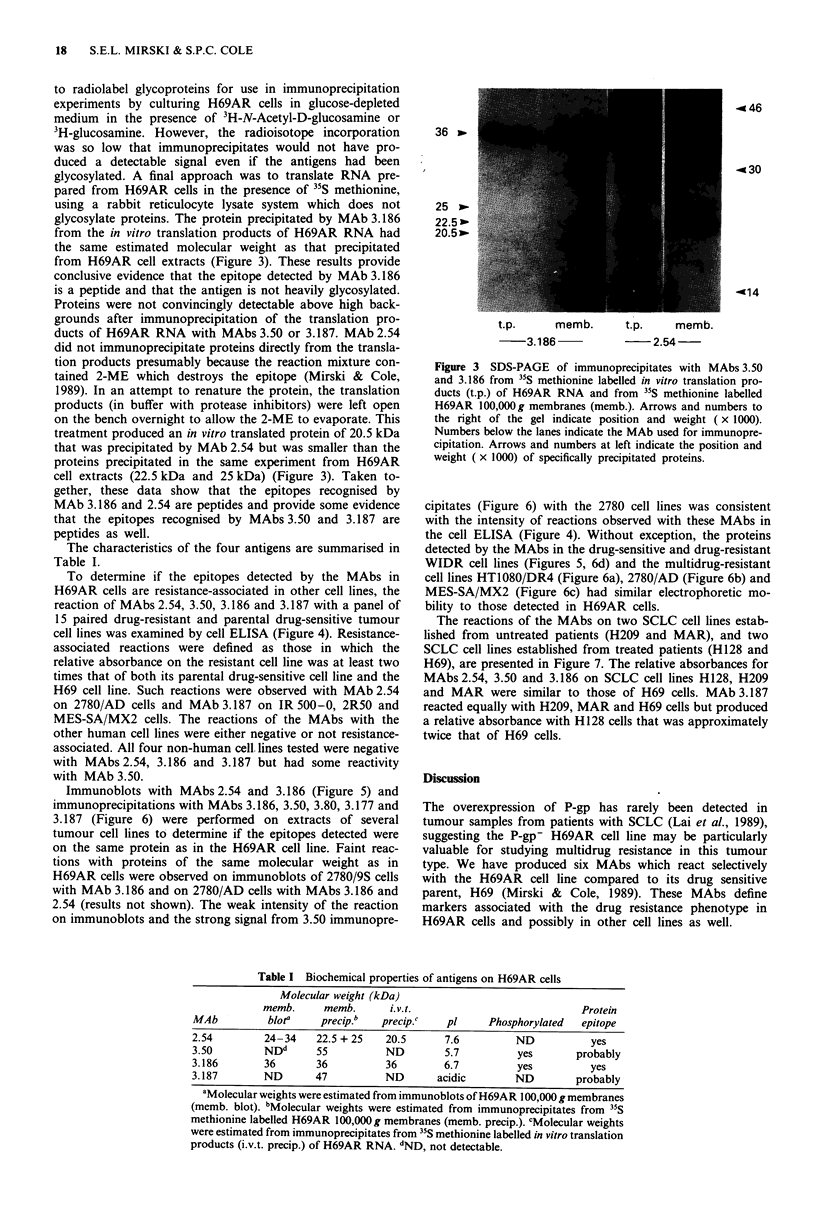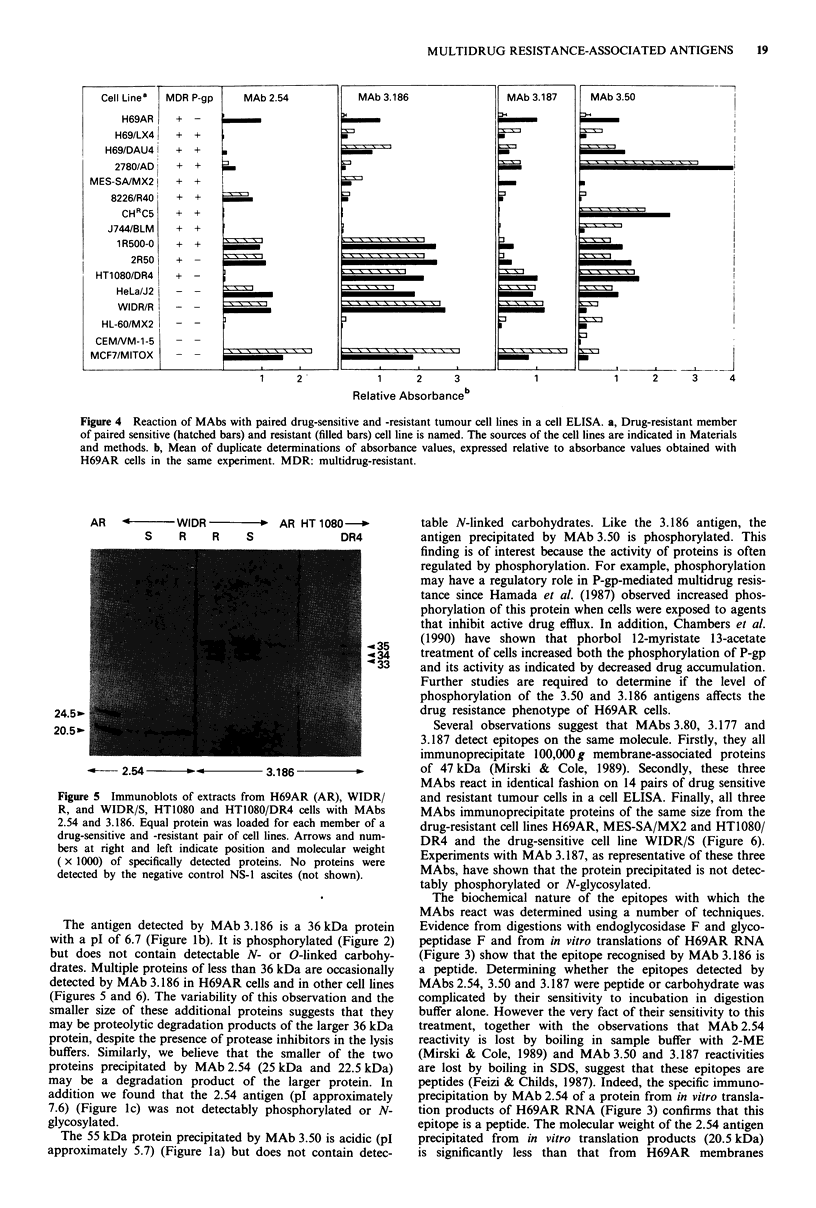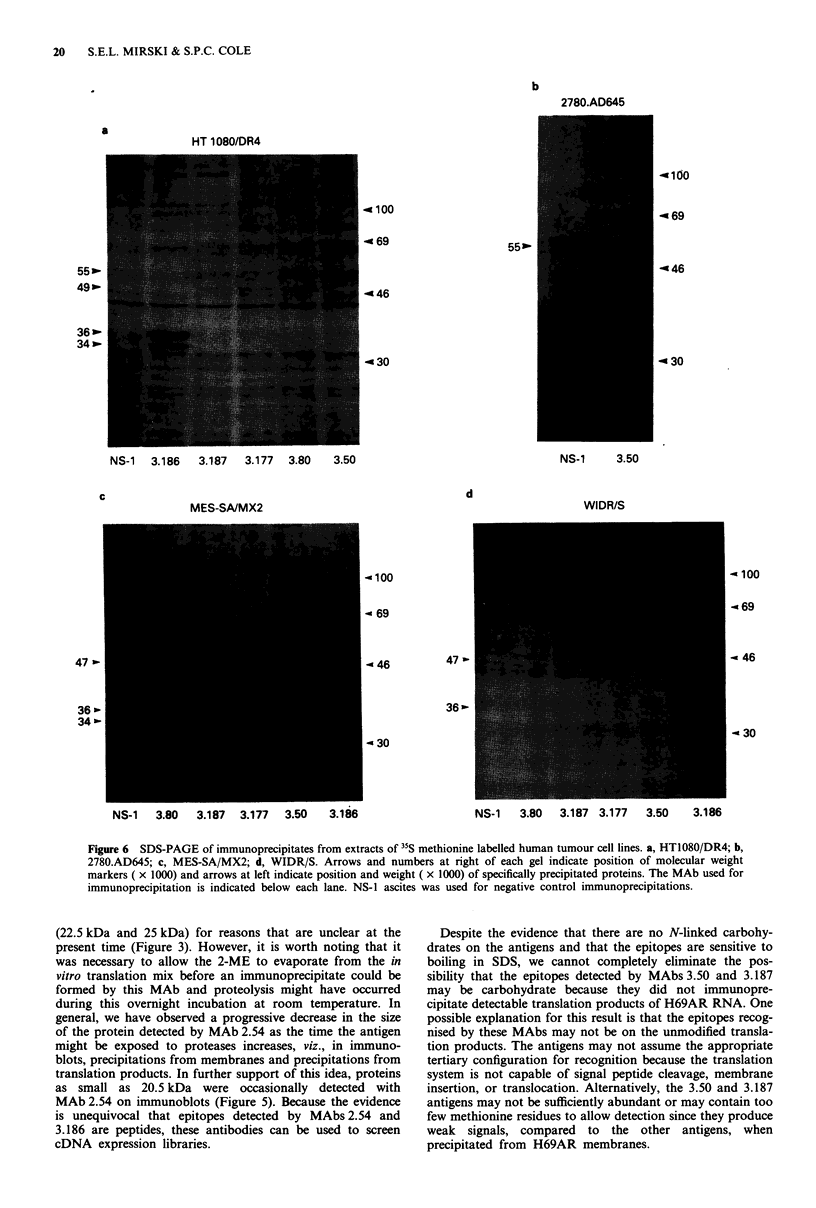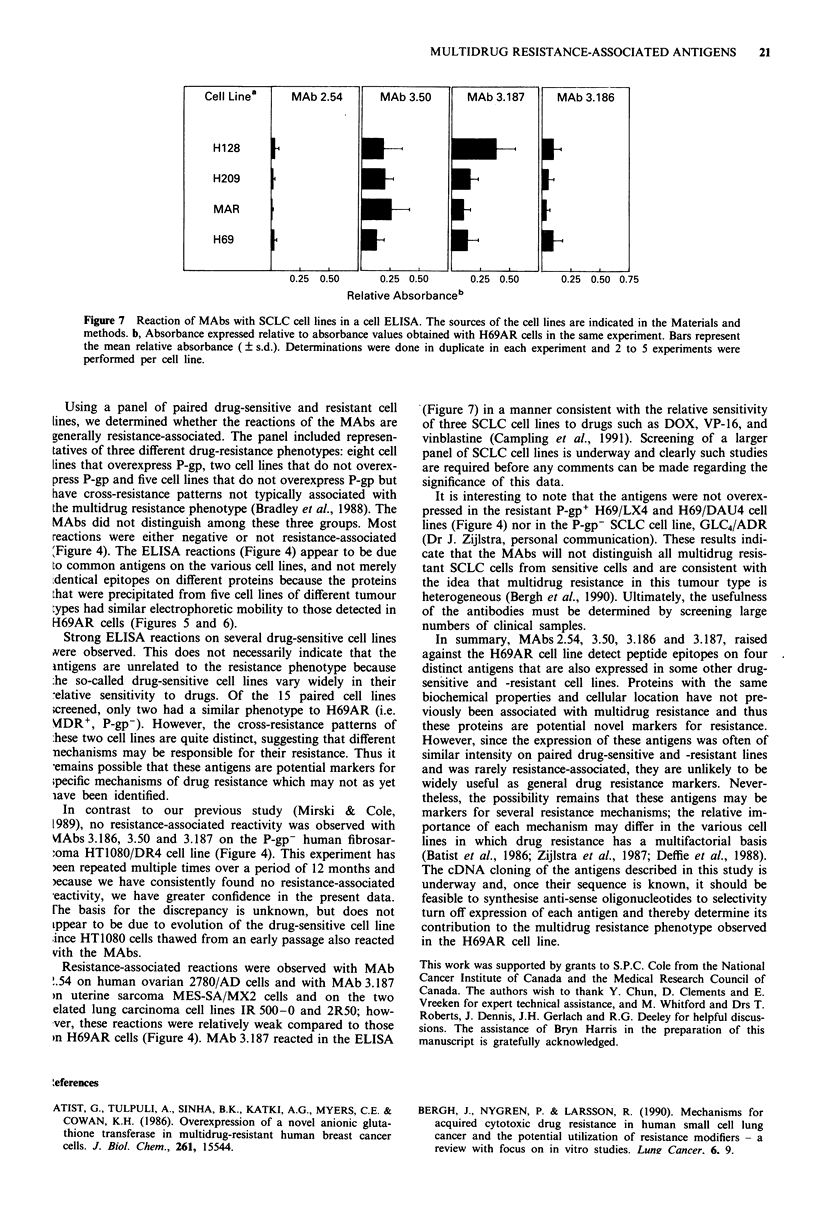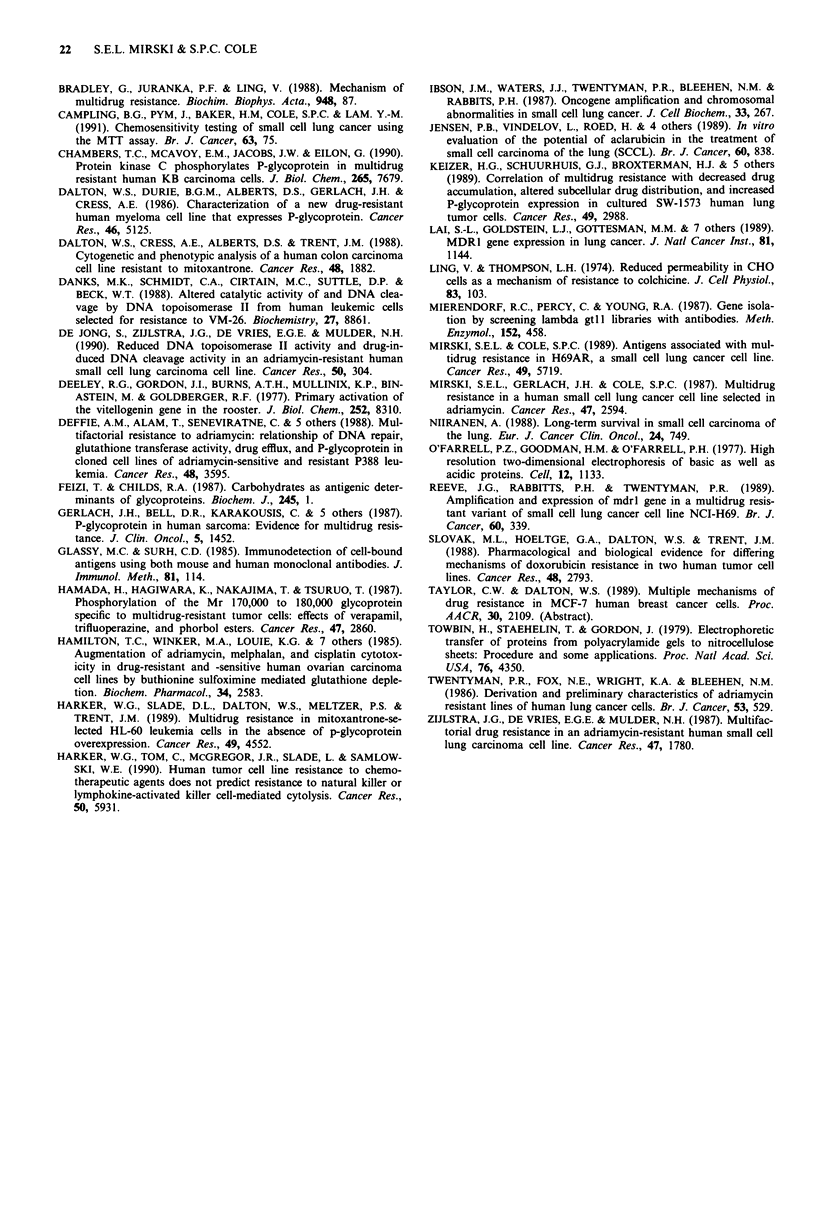# Multidrug resistance-associated antigens on drug-sensitive and -resistant human tumour cell lines.

**DOI:** 10.1038/bjc.1991.232

**Published:** 1991-07

**Authors:** S. E. Mirski, S. P. Cole

**Affiliations:** Department of Oncology, Queen's University, Kingston, Ontario, Canada.

## Abstract

**Images:**


### Multidrug resistance-associated antigens on drug-sensitive and -resistant human tumour cell lines

S.E.L. Mirski[1] & S.P.C. Cole[1,2,3]

*Departments of [1]Oncology and [2]Pharmacology & Toxicology, Queen's University, Kingston, Ontario, K7L 3N6 Canada; and [3]Ontario Cancer Treatment and Research Foundation, Kingston Regional Cancer Centre, Kingston, Ontario, K7L 2V7 Canada.*

**Summary** In this paper the biochemical properties of the antigens detected by six murine monoclonal antibodies (MAbs) are described. These MAbs react selectively with the multidrug-resistant small cell lung cancer (SCLC) cell line, H69AR, compared to its sensitive parent cell line, H69 (Mirski & Cole, 1989). Because H69AR cells do not overexpress P-glycoprotein, the antigens detected by these MAbs may be markers for non-P-glycoprotein-mediated mechanisms of resistance. We found that the 36 kDa protein precipitated by MAb 3.186 is phosphorylated and has a pI of approximately 6.7. The 55 kDa protein precipitated by MAb 3.50 is also phosphorylated and has a pI of approximately 5.7. Several observations suggest that MAbs 3.80, 3.177 and 3.187 recognise the same 47 kDa molecule and hence only MAb 3.187 was characterised further. This MAb precipitates an acidic protein which runs as a streak on isoelectric focusing gels. The 25 and 22.5 kDa cell surface proteins precipitated by MAb 2.54 both have a pI of approximately 7.6. Treatment of immunoprecipitates with glycosidase F indicated that none of the proteins detected by MAbs 2.54, 3.187, 3.50 and 3.186 have large *N*-linked carbohydrates. The peptide nature of the epitopes detected by MAbs 2.54 and 3.186 was unequivocally demonstrated by precipitation from *in vitro* translation products of H69AR RNA. The antigens detected by MAbs 3.50 and 3.187 were not detectable in immunoprecipitates of translation products but the epitopes are probably peptides because they were destroyed by boiling in sodium dodecyl sulphate.

When the reaction of the MAbs with a panel of 15 paired drug-sensitive and -resistant cell lines was examined in a cell enzyme-linked immunosorbent assay, only a few resistance associated reactions were observed. Most of the reactions were either negative or not resistance-associated. When tested with three SCLC cell lines, MAb 3.187 reacted in a manner consistent with the relative resistance of the cell lines. Antigens that had similar electrophoretic mobility to those from H69AR cells were precipitated from extracts of five human cell lines of various tumour types. These data indicate that the cross-reactivities of the MAbs are due to antigens shared among the cell lines and not just the expression of common epitopes on different proteins. Resistance-associated proteins with the biochemical properties of the antigens described in this paper have not been reported previously and they remain potential markers for the as yet to be determined mechanisms of drug resistance in SCLC and other human malignancies.

In most patients with small cell lung cancer (SCLC) the effectiveness of chemotherapy is limited by the development of multidrug resistance (Niiranen, 1988). Tissue culture models for this clinical problem include four adriamycin (ADM)-selected multidrug-resistant SCLC cell lines, H69AR, H69/LX4, H69/DAU4 and GLC4/ADR (Mirski *et al.*, 1987; Twentyman *et al.*, 1986; Jensen *et al.*, 1989; Zijlstra *et al.*, 1987). Two of these cell lines, H69/LX4 (Twentyman *et al.*, 1986) and H69/DAU4 (Jensen *et al.*, 1989), overexpress P-glycoprotein (P-gp$^+$) (Reeve *et al.*, 1989), a plasma membrane protein which confers drug resistance by acting as an energy-dependent drug efflux pump to reduce the intracellular drug concentration (Bradley *et al.*, 1988). However, P-gp has been detected infrequently in SCLC tumours that have developed resistance to chemotherapy, suggesting that other mechanisms of resistance are likely to be more important in this disease (Lai *et al.*, 1989). The H69AR (Mirski *et al.*, 1987) and $GLC_4$/ADR (de Jong *et al.*, 1990) cell lines, which do not overexpress P-gp (P-gp$^-$), may therefore be particularly relevant to the study of multidrug resistance in SCLC. The molecular basis of the drug resistance in the H69AR cell line is still uncertain but is probably multifactorial. It is known, however, that this cell line differs from the $GLC_4$/ADR cell line because H69AR cells do not exhibit a drug accumulation defect (unpublished observation).

We have previously described six monoclonal antibodies (MAbs) that detect proteins that might be involved in the mechanism(s) of H69AR cell drug resistance (Mirski & Cole, 1989). One of these antibodies, MAb 2.54, detects a cell surface epitope but does not affect the ADM sensitivity of H69AR cells (unpublished observation). It reacts with multiple proteins of 24.5-34.5 kDa on immunoblots and immunoprecipitates two proteins of 22.5 and 25.5 kDa. Non-cell surface, 100,000 × g membrane-associated epitopes are detected by the other five antibodies, MAbs 3.50, 3.80, 3.177, 3.187 and 3.186. MAbs 3.50 and 3.186 immunoprecipitate antigens of 55 kDa and 36 kDa, respectively, while MAbs 3.80, 3.177 and 3.187 all precipitate a 47 kDa protein. In this paper we present a further biochemical characterisation of the antigens with which these MAbs react in H69AR cells; in addition, we have examined the reaction of the MAbs with a panel of paired drug-sensitive and -resistant cell lines to determine whether these antigens are generally associated with multidrug resistance.

### Materials and methods

### *Cell culture*

The SCLC cell lines, H69, H128 and H209 were provided by Drs A. Gazdar and J. Minna (NIH, Bethesda, MD). The MAR SCLC cell line was provided by Prof A.M. Neville (Ludwig Institute, London, UK) (Ibson *et al.*, 1987). The P-gp$^-$ multidrug-resistant variant of H69, H69AR, was obtained by stepwise selection in ADM and has been described previously (Mirski *et al.*, 1987). These cell lines were cultured in RPMI 1640 medium supplemented with either 5% foetal bovine serum (FBS) (GIBCO Laboratories, Burlington, Ont) or 5% defined/supplemented bovine calf serum (Hyclone Laboratories, Logan, UT). The H69/DAU4 cell line is a daunorubicin-selected multidrug-resistant variant of the human SCLC cell line H69 (Jensen *et al.*, 1989) provided by Dr P.

Correspondence: S.E.L. Mirski, Cancer Research Laboratories, Room 331, Botterell Hall, Queen's University, Kingston, Ontario, Canada K7L 3N6.
Received 20 December 1990; and in revised form 25 February 1991.

Jensen (Finsen Institute, Copenhagen, Denmark). The H69/LX4 cell line is an ADM-selected multidrug resistant variant of the human SCLC cell line H69 (Reeve *et al.*, 1989) provided by Dr P. Twentyman (MRC, Cambridge, UK). The 2R50 and IR500-0 cell lines are ADM-selected multidrug-resistant variants of the human squamous lung carcinoma cell line SW1573 (Keizer *et al.*, 1989). The 2R50 cell line is P-gp$^-$ while the IR500-0 cell line is P-gp$^+$ (Dr F. Baas, Netherlands Cancer Institute, Amsterdam, personal communication). These cell lines were cultured in Ham's F-10 medium (Sigma Chemical Co., St. Louis, MO) supplemented with 10% FBS. HeLa-J2 is an ADM-selected P-gp$^-$ drug-resistant variant of the human cervical carcinoma cell line HeLa-A6, provided by Dr R.M. Baker (Roswell Park Memorial Institute, Buffalo, NY; personal communication). These cell lines were cultured in alpha-minimal essential medium (α-MEM) (GIBCO) with 10% FBS. The MCF-7/MITOX cell line is a mitoxantrone-selected drug-resistant variant of the human breast cancer cell line, MCF-7, provided by Dr W.S. Dalton (Arizona Cancer Center, Tucson, AZ) (Taylor & Dalton, 1989). The 2780/AD cell line is an ADM-selected multidrug-resistant variant of the human ovarian carcinoma cell line A2780/9S (Hamilton *et al.*, 1985). The MES-SA/MX2 cell line is a mitoxantrone-selected P-gp$^+$ multidrug-resistant variant of the human uterine fibrosarcoma cell line MES-SA provided by Dr W.G. Harker (University of Utah, Salt Lake City, UT) (Harker *et al.*, 1990). The HT1080/DR4 cell line is an ADM-selected P-gp$^-$ multidrug-resistant variant of the human fibrosarcoma cell line HT1080 (Slovak *et al.*, 1988). The 8226/R40 cell line is an ADM-selected multidrug-resistant variant of the human myeloma cell line 8226 (Dalton *et al.*, 1986). The HL-60/MX2 cell line is a mitoxantrone-selected drug-resistant variant of the human leukaemia cell line HL-60 (Harker *et al.*, 1989). The CEM/VM-1-5 cell line is a VM-26-selected drug-resistant variant of the human leukaemia cell line CEM (Danks *et al.*, 1988). The WIDR/R cell line is a mitoxantrone-selected drug-resistant variant of the human colon carcinoma cell line WIDR/S (Dalton *et al.*, 1988). The CH$^R$C5 cell line is a colchicine-selected multidrug-resistant variant of the Chinese hamster ovary cell line CHO/AUXB1 (Ling & Thompson, 1974). The murine macrophage-like cell line J744.2 and its bleomycin-selected multidrug-resistant variant were provided by Dr S. Horwitz (Albert Einstein College of Medicine, New York, NY) and maintained in Dulbecco's modified Eagle's medium with 10% FBS and 1 mM non-essential amino acids.

Unless otherwise indicated, drug-resistant cell lines and their drug-sensitive parental cell lines were obtained directly from the laboratories of origin and cultured according to the conditions described in the references cited. All cell lines were cultured at 37°C in 95% air, 5% $CO_2$ atmosphere and were free of mycoplasma contamination.

*Monoclonal antibodies*

The production of MAbs 2.54, 3.50, 3.186, 3.80, 3.177 and 3.187 has been described previously (Mirski & Cole, 1989). Spleen cells from BALB/c mice which had received multiple injections of viable H69AR cells, were fused with P3.NS1/Ag4.1 (NS-1) myeloma cells and hybridomas were selected which (i) reacted with H69 cells similar to negative control (NS-1 ascites) values in a cell enzyme-linked immunosorbent assay (ELISA), (ii) had a ratio of ELISA absorbance values (H69AR: H69) greater than 5, and (iii) had consistent specific reactivity with H69AR cells following cloning, expansion and freezing.

*Enzyme-linked immunosorbent assay*

Binding of the MAbs to monolayers of tumours cells was assessed by a modification of the method of Glassy and Surh (1985) as described previously (Mirski & Cole, 1989). Briefly, $5 \times 10^4$ cells per well, in 96 well polyvinyl chloride plates (Falcon 3912, Becton-Dickinson, Oxnard, CA), were dried overnight at 37°C and used immediately or stored at 4°C and used within 1 week. Hybridoma culture supernatant was added at 1:5 final dilution or ascites at 1:250 final dilution in a blocking solution of 1% bovine serum albumin (BSA), 5% normal goat serum (NGS) in phosphate-buffered saline (PBS). Binding of the MAb was detected using horseradish peroxidase-conjugated goat anti-mouse Ig(G + M + A) affinity purified F(ab')$_2$ fragments (Cappel, Cooper Biomedical, Malvern, PA) with o-phenylenediamine and hydrogen peroxide as substrates. Colour development was measured on a Dynatech MR600 microtitre plate reader.

*Immunoblotting*

Cells were homogenised in 10 mM Tris-HCl, pH 7.6 buffer containing 10 mM KCl, 1.5 mM $MgCl_2$, 2 mM phenylmethylsulfonyl fluoride (PMSF) and 0.5% Aprotinin (Sigma) at 4°C, and 100,000 *g* membranes were prepared by the method of Gerlach *et al.* (1987). Cell membrane preparations in sample buffer without 2-mercaptoethanol (2-ME) were run on sodium dodecyl sulfate-polyacrylamide gels (SDS-PAGE) and replica blotted onto Immobolin (Millipore, Mississauga, Ont) by the method of Towbin *et al.* (1979). The blot was blocked with 5% FBS/5% NGS/0.05% Tween 20 in PBS (blocking buffer) and then incubated with MAb 2.54 or MAb 3.186 or NS-1 negative control ascites diluted 1:500 in blocking buffer. Binding of the MAbs was detected using alkaline phosphatase-conjugated goat anti-mouse IgG (Jackson ImmunoResearch Laboratories, West Grove, PA) with nitroblue tetrazolium and bromochloroindolyl phosphate (Sigma) as substrates (Mierendorf *et al.*, 1987).

*Immunoprecipitations*

MAb ascites, rabbit anti-mouse Igs (DAKO Immunoglobulins, Denmark) and *S.aureus* (Sigma) were used to immunoprecipitate antigens, as previously described (Mirski & Cole, 1989), from 100,000 *g* membranes or crude extracts prepared from cells which had been labelled in tissue culture. Alternatively, to reduce non-specific precipitation, protein A Sepharose CL-4B (Pharmacia, Balie d'Urfé, Que) was used and was incubated with rabbit anti-mouse Igs, and MAb ascites and washed prior to use. Immunoprecipitates were washed, boiled 5 min in sample buffer with 2-ME, microfuged, and the supernatant loaded for SDS-PAGE.

Cells were cultured in the presence of 30 $\mu$Ci ml$^{-1}$ $^{35}$S methionine (cell labelling grade, 640-700 Ci mmol$^{-1}$) (DuPont, Mississauga, Ont) for 24 h in methionine-deficient RPMI 1640 (Sigma) with 10% dialysed FBS or with 750 $\mu$Ci ml$^{-1}$ $^{32}PO_4$ (8500-9210 Ci mmol$^{-1}$) (DuPont) in phosphate-deficient Minimum Essential Medium, Eagle (modified) with Earle's salts (Flow Laboratories, McLean, VA) and 10% dialysed FBS for 2.5 h. An aliquot of 100,000 *g* membrane containing approximately $2 \times 10^6$ acid-precipitable c.p.m. of $^{35}$S or $4 \times 10^5$ acid-precipitable c.p.m. of $^{32}$P was immunoprecipitated.

In some experiments, immunoprecipitates were used for two dimensional electrophoresis as described by O'Farrell *et al.* (1977). The first dimension was isoelectric focusing with pH 3 to 10 Ampholines (Pharmacia) or non-equilibrium pH gradient electrophoresis (NEPHGE) in which the pH gradient is reversed. The second dimension was SDS-PAGE. Carbamylated protein standards (Pharmacia) were used to estimate pI. Gels were fixed, those containing $^{35}$S were soaked in Amplify (Amersham, Oakville, Ont) and dried. Autoradiographs were obtained with Kodak X-AR film at $-70$°C.

*Enzyme digestions*

A glycosidase F preparation containing endoglycosidase F and glycopeptidase F in a 1:1 ratio (Boehringer Mannheim, Indianapolis, IN) was used to hydrolyse hybrid and complex oligosaccharides using conditions recommended by the supplier. Briefly, proteins were denatured by boiling for 3 min with SDS (SDS:protein ratio of 1:1 by weight), and the sample diluted so that the final concentration of SDS in the incubation mix was approximately 1 mg ml$^{-1}$ in 0.25 M Na acetate, pH 7 with 0.01% $NaN_3$, 6 mg ml$^{-1}$ NP-40, 20 mM EDTA, 10 mM 2-ME, 1 mM PMSF, and 0.5% Aprotinin. Endoglycosidase F was added to a final concentration of 3.4 to 3.6 units ml$^{-1}$, and incubated at 37°C for 17 to 19 h. Negative control digestions were performed in the absence of enzyme. For some experiments 100,000 *g* membrane preparations of H69AR cells which had been labelled in culture with $^{35}$S methionine were digested with the endoglycosidase F preparation and then immunoprecipitated with the MAbs. In other experiments digestions were performed on immunoprecipitates. Digests of fetuin were included in both types of experiment and the increase in its electrophoretic mobility was monitored as a positive control for enzyme activity.

### In vitro *translations*

Total RNA prepared from H69AR cells by the guanidine-HCl/sodium acetate method of Deeley *et al.* (1977) was translated *in vitro* in the presence of 0.3 mCi $^{35}$S methionine ml$^{-1}$ (translation grade, 1146 Ci mmol$^{-1}$) (DuPont) using a rabbit reticulocyte lysate kit (Promega, Madison, WI) and a final RNA concentration of 200 μg ml$^{-1}$. Immunoprecipitates from $2.4 \times 10^6$ to $6.0 \times 10^6$ acid-precipitable c.p.m. of the *in vitro* translation products were processed for SDS-PAGE and autoradiography as described above.

### Results

We have previously shown that MAbs 3.187, 3.177, and 3.80 all precipitate proteins of 47 kDa, suggesting that they may detect epitopes on the same protein (Mirski & Cole, 1989). In this study, the cell ELISA reaction pattern of each of these MAbs was the same in all 14 pairs of drug-sensitive and -resistant tumour cell lines tested (data not shown), providing additional evidence that these three MAbs likely detect epitopes on the same molecule. For this reason, MAb 3.187 was selected as a representative of this group for use in most subsequent experiments.

Two-dimensional gel electrophoresis showed that the antigen precipitated by MAb 3.50 is acidic (approximate pI 5.7) (Figure 1a). The antigen precipitated by MAb 3.187 was also acidic, but a pI could not be determined because the protein ran as a streak (data not shown). The immunoprecipitates obtained with MAbs 3.186 and 2.54 were run on NEPHGE to obtain an estimate of pIs which were approximately 6.7 and 7.6 respectively (Figure 1b and 1c).

To determine if the proteins detected by the MAbs are phosphorylated, immunoprecipitations were performed on membrane preparations from H69AR cells that had been labelled in culture with $^{32}PO_4$. As shown in Figure 2, the molecules detected by MAbs 3.50 and 3.186 are phosphorylated but phosphorylation of the other antigens were not detected.

Several approaches were taken to determine if the antigens detected in H69AR cells are glycosylated and whether the epitopes detected are peptide or carbohydrate in nature. First, H69AR membrane preparations were digested with endoglycosidase F and glycopeptidase F and immunoprecipitated. MAb 3.186 precipitated a protein of the same electrophoretic mobility from digested or control H69AR membranes, suggesting that the epitope detected is not an *N*-linked carbohydrate and that the antigen is not extensively glycosylated (results not shown). Results from digestions with endoglycosidases prior to immunoprecipitation were not informative for MAbs 2.54, 3.50 and 3.187 because the epitopes were destroyed in the control digests without enzyme. Control digests required boiling of the cell extract in SDS. This resulted in the loss of antigenicity detectable by MAbs 3.50 and 3.187, suggesting that these epitopes are peptides (Feizi & Childs, 1987). The inclusion of 2-ME caused the loss of MAb 2.54 antigenicity. In a second approach, $^{35}$S methionine-labelled immunoprecipitates were digested with endoglycosidase F and glycopeptidase F and no change in the size of the proteins was seen, suggesting that large *N*-linked carbohydrates are not present on these antigens (results not shown). In a third approach, we attempted

**Figure 1** Two-dimensional gel electrophoresis of immunoprecipitates from 100,000 *g* membrane preparations of $^{35}$S methionine labelled H69AR cells **a**, MAb 3.50, **b**, MAb 3.186 and **c**, MAb 2.54. Numbers and arrows below the gel indicate the specifically immunoprecipitated protein. Arrows and numbers to the right of the gels indicate the position of molecular weight markers ( × 1000).

**Figure 2** SDS-PAGE of immunoprecipitates with MAbs 3.50 and 3.186 from 100,000 *g* membrane preparations of $^{32}PO_4$ labelled H69AR cells. Arrows and numbers to the left of the gel indicate the position and molecular weight ( × 1000) of specifically precipitated proteins. Arrows and numbers to the right of the gel indicate position of molecular weight markers ( × 1000).

to radiolabel glycoproteins for use in immunoprecipitation experiments by culturing H69AR cells in glucose-depleted medium in the presence of $^{3}$H-*N*-Acetyl-D-glucosamine or $^{3}$H-glucosamine. However, the radioisotope incorporation was so low that immunoprecipitates would not have produced a detectable signal even if the antigens had been glycosylated. A final approach was to translate RNA prepared from H69AR cells in the presence of $^{35}$S methionine, using a rabbit reticulocyte lysate system which does not glycosylate proteins. The protein precipitated by MAb 3.186 from the *in vitro* translation products of H69AR RNA had the same estimated molecular weight as that precipitated from H69AR cell extracts (Figure 3). These results provide conclusive evidence that the epitope detected by MAb 3.186 is a peptide and that the antigen is not heavily glycosylated. Proteins were not convincingly detectable above high backgrounds after immunoprecipitation of the translation products of H69AR RNA with MAbs 3.50 or 3.187. MAb 2.54 did not immunoprecipitate proteins directly from the translation products presumably because the reaction mixture contained 2-ME which destroys the epitope (Mirski & Cole, 1989). In an attempt to renature the protein, the translation products (in buffer with protease inhibitors) were left open on the bench overnight to allow the 2-ME to evaporate. This treatment produced an *in vitro* translated protein of 20.5 kDa that was precipitated by MAb 2.54 but was smaller than the proteins precipitated in the same experiment from H69AR cell extracts (22.5 kDa and 25 kDa) (Figure 3). Taken together, these data show that the epitopes recognised by MAb 3.186 and 2.54 are peptides and provide some evidence that the epitopes recognised by MAbs 3.50 and 3.187 are peptides as well.

The characteristics of the four antigens are summarised in Table I.

To determine if the epitopes detected by the MAbs in H69AR cells are resistance-associated in other cell lines, the reaction of MAbs 2.54, 3.50, 3.186 and 3.187 with a panel of 15 paired drug-resistant and parental drug-sensitive tumour cell lines was examined by cell ELISA (Figure 4). Resistance-associated reactions were defined as those in which the relative absorbance on the resistant cell line was at least two times that of both its parental drug-sensitive cell line and the H69 cell line. Such reactions were observed with MAb 2.54 on 2780/AD cells and MAb 3.187 on IR 500-0, 2R50 and MES-SA/MX2 cells. The reactions of the MAbs with the other human cell lines were either negative or not resistance-associated. All four non-human cell lines tested were negative with MAbs 2.54, 3.186 and 3.187 but had some reactivity with MAb 3.50.

Immunoblots with MAbs 2.54 and 3.186 (Figure 5) and immunoprecipitations with MAbs 3.186, 3.50, 3.80, 3.177 and 3.187 (Figure 6) were performed on extracts of several tumour cell lines to determine if the epitopes detected were on the same protein as in the H69AR cell line. Faint reactions with proteins of the same molecular weight as in H69AR cells were observed on immunoblots of 2780/9S cells with MAb 3.186 and on 2780/AD cells with MAbs 3.186 and 2.54 (results not shown). The weak intensity of the reaction on immunoblots and the strong signal from 3.50 immunoprecipitates (Figure 6) with the 2780 cell lines was consistent with the intensity of reactions observed with these MAbs in the cell ELISA (Figure 4). Without exception, the proteins detected by the MAbs in the drug-sensitive and drug-resistant WIDR cell lines (Figures 5, 6d) and the multidrug-resistant cell lines HT1080/DR4 (Figure 6a), 2780/AD (Figure 6b) and MES-SA/MX2 (Figure 6c) had similar electrophoretic mobility to those detected in H69AR cells.

**Figure 3** SDS-PAGE of immunoprecipitates with MAbs 3.50 and 3.186 from $^{35}$S methionine labelled *in vitro* translation products (t.p.) of H69AR RNA and from $^{35}$S methionine labelled H69AR 100,000 *g* membranes (memb.). Arrows and numbers to the right of the gel indicate position and weight ( × 1000). Numbers below the lanes indicate the MAb used for immunoprecipitation. Arrows and numbers at left indicate the position and weight ( × 1000) of specifically precipitated proteins.

The reactions of the MAbs on two SCLC cell lines established from untreated patients (H209 and MAR), and two SCLC cell lines established from treated patients (H128 and H69), are presented in Figure 7. The relative absorbances for MAbs 2.54, 3.50 and 3.186 on SCLC cell lines H128, H209 and MAR were similar to those of H69 cells. MAb 3.187 reacted equally with H209, MAR and H69 cells but produced a relative absorbance with H128 cells that was approximately twice that of H69 cells.

### Discussion

The overexpression of P-gp has rarely been detected in tumour samples from patients with SCLC (Lai *et al.*, 1989), suggesting the P-gp$^{-}$ H69AR cell line may be particularly valuable for studying multidrug resistance in this tumour type. We have produced six MAbs which react selectively with the H69AR cell line compared to its drug sensitive parent, H69 (Mirski & Cole, 1989). These MAbs define markers associated with the drug resistance phenotype in H69AR cells and possibly in other cell lines as well.

**Table I** Biochemical properties of antigens on H69AR cells

| *MAb* | *Molecular weight (kDa)* *memb. blot*[a] | *memb. precip.*[b] | *i.v.t. precip.*[c] | *pI* | *Phosphorylated* | *Protein epitope* |
|---|---|---|---|---|---|---|
| 2.54 | 24–34 | 22.5 + 25 | 20.5 | 7.6 | ND | yes |
| 3.50 | ND[d] | 55 | ND | 5.7 | yes | probably |
| 3.186 | 36 | 36 | 36 | 6.7 | yes | yes |
| 3.187 | ND | 47 | ND | acidic | ND | probably |

[a]Molecular weights were estimated from immunoblots of H69AR 100,000 *g* membranes (memb. blot). [b]Molecular weights were estimated from immunoprecipitates from $^{35}$S methionine labelled H69AR 100,000 *g* membranes (memb. precip.). [c]Molecular weights were estimated from immunoprecipitates from $^{35}$S methionine labelled *in vitro* translation products (i.v.t. precip.) of H69AR RNA. [d]ND, not detectable.

**Figure 4** Reaction of MAbs with paired drug-sensitive and -resistant tumour cell lines in a cell ELISA. **a**, Drug-resistant member of paired sensitive (hatched bars) and resistant (filled bars) cell line is named. The sources of the cell lines are indicated in Materials and methods. **b**, Mean of duplicate determinations of absorbance values, expressed relative to absorbance values obtained with H69AR cells in the same experiment. MDR: multidrug-resistant.

**Figure 5** Immunoblots of extracts from H69AR (AR), WIDR/R, and WIDR/S, HT1080 and HT1080/DR4 cells with MAbs 2.54 and 3.186. Equal protein was loaded for each member of a drug-sensitive and -resistant pair of cell lines. Arrows and numbers at right and left indicate position and molecular weight ( × 1000) of specifically detected proteins. No proteins were detected by the negative control NS-1 ascites (not shown).

The antigen detected by MAb 3.186 is a 36 kDa protein with a pI of 6.7 (Figure 1b). It is phosphorylated (Figure 2) but does not contain detectable *N*- or *O*-linked carbohydrates. Multiple proteins of less than 36 kDa are occasionally detected by MAb 3.186 in H69AR cells and in other cell lines (Figures 5 and 6). The variability of this observation and the smaller size of these additional proteins suggests that they may be proteolytic degradation products of the larger 36 kDa protein, despite the presence of protease inhibitors in the lysis buffers. Similarly, we believe that the smaller of the two proteins precipitated by MAb 2.54 (25 kDa and 22.5 kDa) may be a degradation product of the larger protein. In addition we found that the 2.54 antigen (pI approximately 7.6) (Figure 1c) was not detectably phosphorylated or *N*-glycosylated.

The 55 kDa protein precipitated by MAb 3.50 is acidic (pI approximately 5.7) (Figure 1a) but does not contain detectable *N*-linked carbohydrates. Like the 3.186 antigen, the antigen precipitated by MAb 3.50 is phosphorylated. This finding is of interest because the activity of proteins is often regulated by phosphorylation. For example, phosphorylation may have a regulatory role in P-gp-mediated multidrug resistance since Hamada *et al.* (1987) observed increased phosphorylation of this protein when cells were exposed to agents that inhibit active drug efflux. In addition, Chambers *et al.* (1990) have shown that phorbol 12-myristate 13-acetate treatment of cells increased both the phosphorylation of P-gp and its activity as indicated by decreased drug accumulation. Further studies are required to determine if the level of phosphorylation of the 3.50 and 3.186 antigens affects the drug resistance phenotype of H69AR cells.

Several observations suggest that MAbs 3.80, 3.177 and 3.187 detect epitopes on the same molecule. Firstly, they all immunoprecipitate 100,000 *g* membrane-associated proteins of 47 kDa (Mirski & Cole, 1989). Secondly, these three MAbs react in identical fashion on 14 pairs of drug sensitive and resistant tumour cells in a cell ELISA. Finally, all three MAbs immunoprecipitate proteins of the same size from the drug-resistant cell lines H69AR, MES-SA/MX2 and HT1080/DR4 and the drug-sensitive cell line WIDR/S (Figure 6). Experiments with MAb 3.187, as representative of these three MAbs, have shown that the protein precipitated is not detectably phosphorylated or *N*-glycosylated.

The biochemical nature of the epitopes with which the MAbs react was determined using a number of techniques. Evidence from digestions with endoglycosidase F and glycopeptidase F and from *in vitro* translations of H69AR RNA (Figure 3) show that the epitope recognised by MAb 3.186 is a peptide. Determining whether the epitopes detected by MAbs 2.54, 3.50 and 3.187 were peptide or carbohydrate was complicated by their sensitivity to incubation in digestion buffer alone. However the very fact of their sensitivity to this treatment, together with the observations that MAb 2.54 reactivity is lost by boiling in sample buffer with 2-ME (Mirski & Cole, 1989) and MAb 3.50 and 3.187 reactivities are lost by boiling in SDS, suggest that these epitopes are peptides (Feizi & Childs, 1987). Indeed, the specific immunoprecipitation by MAb 2.54 of a protein from *in vitro* translation products of H69AR RNA (Figure 3) confirms that this epitope is a peptide. The molecular weight of the 2.54 antigen precipitated from *in vitro* translation products (20.5 kDa) is significantly less than that from H69AR membranes

**Figure 6** SDS-PAGE of immunoprecipitates from extracts of $^{35}S$ methionine labelled human tumour cell lines. **a**, HT1080/DR4; **b**, 2780.AD645; **c**, MES-SA/MX2; **d**, WIDR/S. Arrows and numbers at right of each gel indicate position of molecular weight markers ( × 1000) and arrows at left indicate position and weight ( × 1000) of specifically precipitated proteins. The MAb used for immunoprecipitation is indicated below each lane. NS-1 ascites was used for negative control immunoprecipitations.

(22.5 kDa and 25 kDa) for reasons that are unclear at the present time (Figure 3). However, it is worth noting that it was necessary to allow the 2-ME to evaporate from the *in vitro* translation mix before an immunoprecipitate could be formed by this MAb and proteolysis might have occurred during this overnight incubation at room temperature. In general, we have observed a progressive decrease in the size of the protein detected by MAb 2.54 as the time the antigen might be exposed to proteases increases, *viz*., in immunoblots, precipitations from membranes and precipitations from translation products. In further support of this idea, proteins as small as 20.5 kDa were occasionally detected with MAb 2.54 on immunoblots (Figure 5). Because the evidence is unequivocal that epitopes detected by MAbs 2.54 and 3.186 are peptides, these antibodies can be used to screen cDNA expression libraries.

Despite the evidence that there are no *N*-linked carbohydrates on the antigens and that the epitopes are sensitive to boiling in SDS, we cannot completely eliminate the possibility that the epitopes detected by MAbs 3.50 and 3.187 may be carbohydrate because they did not immunoprecipitate detectable translation products of H69AR RNA. One possible explanation for this result is that the epitopes recognised by these MAbs may not be on the unmodified translation products. The antigens may not assume the appropriate tertiary configuration for recognition because the translation system is not capable of signal peptide cleavage, membrane insertion, or translocation. Alternatively, the 3.50 and 3.187 antigens may not be sufficiently abundant or may contain too few methionine residues to allow detection since they produce weak signals, compared to the other antigens, when precipitated from H69AR membranes.

**Figure 7** Reaction of MAbs with SCLC cell lines in a cell ELISA. The sources of the cell lines are indicated in the Materials and methods. **b**, Absorbance expressed relative to absorbance values obtained with H69AR cells in the same experiment. Bars represent the mean relative absorbance ( ± s.d.). Determinations were done in duplicate in each experiment and 2 to 5 experiments were performed per cell line.

Using a panel of paired drug-sensitive and resistant cell lines, we determined whether the reactions of the MAbs are generally resistance-associated. The panel included representatives of three different drug-resistance phenotypes: eight cell lines that overexpress P-gp, two cell lines that do not overexpress P-gp and five cell lines that do not overexpress P-gp but have cross-resistance patterns not typically associated with the multidrug resistance phenotype (Bradley *et al.*, 1988). The MAbs did not distinguish among these three groups. Most reactions were either negative or not resistance-associated (Figure 4). The ELISA reactions (Figure 4) appear to be due to common antigens on the various cell lines, and not merely identical epitopes on different proteins because the proteins that were precipitated from five cell lines of different tumour types had similar electrophoretic mobility to those detected in H69AR cells (Figures 5 and 6).

Strong ELISA reactions on several drug-sensitive cell lines were observed. This does not necessarily indicate that the antigens are unrelated to the resistance phenotype because the so-called drug-sensitive cell lines vary widely in their relative sensitivity to drugs. Of the 15 paired cell lines screened, only two had a similar phenotype to H69AR (i.e. MDR$^+$, P-gp$^-$). However, the cross-resistance patterns of these two cell lines are quite distinct, suggesting that different mechanisms may be responsible for their resistance. Thus it remains possible that these antigens are potential markers for specific mechanisms of drug resistance which may not as yet have been identified.

In contrast to our previous study (Mirski & Cole, 1989), no resistance-associated reactivity was observed with MAbs 3.186, 3.50 and 3.187 on the P-gp$^-$ human fibrosarcoma HT1080/DR4 cell line (Figure 4). This experiment has been repeated multiple times over a period of 12 months and because we have consistently found no resistance-associated reactivity, we have greater confidence in the present data. The basis for the discrepancy is unknown, but does not appear to be due to evolution of the drug-sensitive cell line since HT1080 cells thawed from an early passage also reacted with the MAbs.

Resistance-associated reactions were observed with MAb 2.54 on human ovarian 2780/AD cells and with MAb 3.187 on uterine sarcoma MES-SA/MX2 cells and on the two related lung carcinoma cell lines IR 500-0 and 2R50; however, these reactions were relatively weak compared to those on H69AR cells (Figure 4). MAb 3.187 reacted in the ELISA (Figure 7) in a manner consistent with the relative sensitivity of three SCLC cell lines to drugs such as DOX, VP-16, and vinblastine (Campling *et al.*, 1991). Screening of a larger panel of SCLC cell lines is underway and clearly such studies are required before any comments can be made regarding the significance of this data.

It is interesting to note that the antigens were not overexpressed in the resistant P-gp$^+$ H69/LX4 and H69/DAU4 cell lines (Figure 4) nor in the P-gp$^-$ SCLC cell line, $GLC_4$/ADR (Dr J. Zijlstra, personal communication). These results indicate that the MAbs will not distinguish all multidrug resistant SCLC cells from sensitive cells and are consistent with the idea that multidrug resistance in this tumour type is heterogeneous (Bergh *et al.*, 1990). Ultimately, the usefulness of the antibodies must be determined by screening large numbers of clinical samples.

In summary, MAbs 2.54, 3.50, 3.186 and 3.187, raised against the H69AR cell line detect peptide epitopes on four distinct antigens that are also expressed in some other drug-sensitive and -resistant cell lines. Proteins with the same biochemical properties and cellular location have not previously been associated with multidrug resistance and thus these proteins are potential novel markers for resistance. However, since the expression of these antigens was often of similar intensity on paired drug-sensitive and -resistant lines and was rarely resistance-associated, they are unlikely to be widely useful as general drug resistance markers. Nevertheless, the possibility remains that these antigens may be markers for several resistance mechanisms; the relative importance of each mechanism may differ in the various cell lines in which drug resistance has a multifactorial basis (Batist *et al.*, 1986; Zijlstra *et al.*, 1987; Deffie *et al.*, 1988). The cDNA cloning of the antigens described in this study is underway and, once their sequence is known, it should be feasible to synthesise anti-sense oligonucleotides to selectivity turn off expression of each antigen and thereby determine its contribution to the multidrug resistance phenotype observed in the H69AR cell line.

This work was supported by grants to S.P.C. Cole from the National Cancer Institute of Canada and the Medical Research Council of Canada. The authors wish to thank Y. Chun, D. Clements and E. Vreeken for expert technical assistance, and M. Whitford and Drs T. Roberts, J. Dennis, J.H. Gerlach and R.G. Deeley for helpful discussions. The assistance of Bryn Harris in the preparation of this manuscript is gratefully acknowledged.

### References

BATIST, G., TULPULI, A., SINHA, B.K., KATKI, A.G., MYERS, C.E. & COWAN, K.H. (1986). Overexpression of a novel anionic glutathione transferase in multidrug-resistant human breast cancer cells. *J. Biol. Chem.*, **261**, 15544.

BERGH, J., NYGREN, P. & LARSSON, R. (1990). Mechanisms for acquired cytotoxic drug resistance in human small cell lung cancer and the potential utilization of resistance modifiers – a review with focus on in vitro studies. *Lung Cancer*, **6**, 9.

BRADLEY, G., JURANKA, P.F. & LING, V. (1988). Mechanism of multidrug resistance. *Biochim. Biophys. Acta.*, **948,** 87.

CAMPLING, B.G., PYM, J., BAKER, H.M, COLE, S.P.C. & LAM. Y.-M. (1991). Chemosensitivity testing of small cell lung cancer using the MTT assay. *Br. J. Cancer*, **63,** 75.

CHAMBERS, T.C., MCAVOY, E.M., JACOBS, J.W. & EILON, G. (1990). Protein kinase C phosphorylates P-glycoprotein in multidrug resistant human KB carcinoma cells. *J. Biol. Chem.*, **265,** 7679.

DALTON, W.S., DURIE, B.G.M., ALBERTS, D.S., GERLACH, J.H. & CRESS, A.E. (1986). Characterization of a new drug-resistant human myeloma cell line that expresses P-glycoprotein. *Cancer Res.*, **46,** 5125.

DALTON, W.S., CRESS, A.E., ALBERTS, D.S. & TRENT, J.M. (1988). Cytogenetic and phenotypic analysis of a human colon carcinoma cell line resistant to mitoxantrone. *Cancer Res.*, **48,** 1882.

DANKS, M.K., SCHMIDT, C.A., CIRTAIN, M.C., SUTTLE, D.P. & BECK, W.T. (1988). Altered catalytic activity of and DNA cleavage by DNA topoisomerase II from human leukemic cells selected for resistance to VM-26. *Biochemistry*, **27,** 8861.

DE JONG, S., ZIJLSTRA, J.G., DE VRIES, E.G.E. & MULDER, N.H. (1990). Reduced DNA topoisomerase II activity and drug-induced DNA cleavage activity in an adriamycin-resistant human small cell lung carcinoma cell line. *Cancer Res.*, **50,** 304.

DEELEY, R.G., GORDON, J.I., BURNS, A.T.H., MULLINIX, K.P., BINASTEIN, M. & GOLDBERGER, R.F. (1977). Primary activation of the vitellogenin gene in the rooster. *J. Biol. Chem.*, **252,** 8310.

DEFFIE, A.M., ALAM, T., SENEVIRATNE, C. & 5 others (1988). Multifactorial resistance to adriamycin: relationship of DNA repair, glutathione transferase activity, drug efflux, and P-glycoprotein in cloned cell lines of adriamycin-sensitive and resistant P388 leukemia. *Cancer Res.*, **48,** 3595.

FEIZI, T. & CHILDS, R.A. (1987). Carbohydrates as antigenic determinants of glycoproteins. *Biochem. J.*, **245,** 1.

GERLACH, J.H., BELL, D.R., KARAKOUSIS, C. & 5 others (1987). P-glycoprotein in human sarcoma: Evidence for multidrug resistance. *J. Clin. Oncol.*, **5,** 1452.

GLASSY, M.C. & SURH, C.D. (1985). Immunodetection of cell-bound antigens using both mouse and human monoclonal antibodies. *J. Immunol. Meth.*, **81,** 114.

HAMADA, H., HAGIWARA, K., NAKAJIMA, T. & TSURUO, T. (1987). Phosphorylation of the Mr 170,000 to 180,000 glycoprotein specific to multidrug-resistant tumor cells: effects of verapamil, trifluoperazine, and phorbol esters. *Cancer Res.*, **47,** 2860.

HAMILTON, T.C., WINKER, M.A., LOUIE, K.G. & 7 others (1985). Augmentation of adriamycin, melphalan, and cisplatin cytotoxicity in drug-resistant and -sensitive human ovarian carcinoma cell lines by buthionine sulfoximine mediated glutathione depletion. *Biochem. Pharmacol.*, **34,** 2583.

HARKER, W.G., SLADE, D.L., DALTON, W.S., MELTZER, P.S. & TRENT, J.M. (1989). Multidrug resistance in mitoxantrone-selected HL-60 leukemia cells in the absence of p-glycoprotein overexpression. *Cancer Res.*, **49,** 4552.

HARKER, W.G., TOM, C., MCGREGOR, J.R., SLADE, L. & SAMLOWSKI, W.E. (1990). Human tumor cell line resistance to chemotherapeutic agents does not predict resistance to natural killer or lymphokine-activated killer cell-mediated cytolysis. *Cancer Res.*, **50,** 5931.

IBSON, J.M., WATERS, J.J., TWENTYMAN, P.R., BLEEHEN, N.M. & RABBITS, P.H. (1987). Oncogene amplification and chromosomal abnormalities in small cell lung cancer. *J. Cell Biochem.*, **33,** 267.

JENSEN, P.B., VINDELOV, L., ROED, H. & 4 others (1989). *In vitro* evaluation of the potential of aclarubicin in the treatment of small cell carcinoma of the lung (SCCL). *Br. J. Cancer*, **60,** 838.

KEIZER, H.G., SCHUURHUIS, G.J., BROXTERMAN, H.J. & 5 others (1989). Correlation of multidrug resistance with decreased drug accumulation, altered subcellular drug distribution, and increased P-glycoprotein expression in cultured SW-1573 human lung tumor cells. *Cancer Res.*, **49,** 2988.

LAI, S.-L., GOLDSTEIN, L.J., GOTTESMAN, M.M. & 7 others (1989). MDR1 gene expression in lung cancer. *J. Natl Cancer Inst.*, **81,** 1144.

LING, V. & THOMPSON, L.H. (1974). Reduced permeability in CHO cells as a mechanism of resistance to colchicine. *J. Cell Physiol.*, **83,** 103.

MIERENDORF, R.C., PERCY, C. & YOUNG, R.A. (1987). Gene isolation by screening lambda gt11 libraries with antibodies. *Meth. Enzymol.*, **152,** 458.

MIRSKI, S.E.L. & COLE, S.P.C. (1989). Antigens associated with multidrug resistance in H69AR, a small cell lung cancer cell line. *Cancer Res.*, **49,** 5719.

MIRSKI, S.E.L., GERLACH, J.H. & COLE, S.P.C. (1987). Multidrug resistance in a human small cell lung cancer cell line selected in adriamycin. *Cancer Res.*, **47,** 2594.

NIIRANEN, A. (1988). Long-term survival in small cell carcinoma of the lung. *Eur. J. Cancer Clin. Oncol.*, **24,** 749.

O'FARRELL, P.Z., GOODMAN, H.M. & O'FARRELL, P.H. (1977). High resolution two-dimensional electrophoresis of basic as well as acidic proteins. *Cell*, **12,** 1133.

REEVE, J.G., RABBITTS, P.H. & TWENTYMAN, P.R. (1989). Amplification and expression of mdr1 gene in a multidrug resistant variant of small cell lung cancer cell line NCI-H69. *Br. J. Cancer*, **60,** 339.

SLOVAK, M.L., HOELTGE, G.A., DALTON, W.S. & TRENT, J.M. (1988). Pharmacological and biological evidence for differing mechanisms of doxorubicin resistance in two human tumor cell lines. *Cancer Res.*, **48,** 2793.

TAYLOR, C.W. & DALTON, W.S. (1989). Multiple mechanisms of drug resistance in MCF-7 human breast cancer cells. *Proc. AACR*, **30,** 2109. (Abstract).

TOWBIN, H., STAEHELIN, T. & GORDON, J. (1979). Electrophoretic transfer of proteins from polyacrylamide gels to nitrocellulose sheets: Procedure and some applications. *Proc. Natl Acad. Sci. USA*, **76,** 4350.

TWENTYMAN, P.R., FOX, N.E., WRIGHT, K.A. & BLEEHEN, N.M. (1986). Derivation and preliminary characteristics of adriamycin resistant lines of human lung cancer cells. *Br. J. Cancer*, **53,** 529.

ZIJLSTRA, J.G., DE VRIES, E.G.E. & MULDER, N.H. (1987). Multifactorial drug resistance in an adriamycin-resistant human small cell lung carcinoma cell line. *Cancer Res.*, **47,** 1780.